# Insight into the Pyrolysis Behaviors of Petroleum-Driven Mesophase Pitch via ReaxFF Molecular Dynamics and In Situ TG-FTIR/MS

**DOI:** 10.3390/ma17215318

**Published:** 2024-10-31

**Authors:** Lingyan Qin, Li Zhao, Bo Yuan, Hongwei Wang, Guojie Liang, Kai Li, Qiang Xie, Lele Gong

**Affiliations:** 1 State Key Laboratory of NBC Protection for Civilian, Beijing 100191, China; 2022200127@buct.edu.cn (L.Q.); zhaolizl2021@163.com (L.Z.); yuanbolanda@163.com (B.Y.); whw13365635280@163.com (H.W.); liangguojie@126.com (G.L.); 750455@sohu.com (K.L.); 2 State Key Laboratory of Organic-Inorganic Composites, College of Chemical Engineering, Beijing University of Chemical Technology, Beijing 100029, China; 3 School of Chemical and Environmental Engineering, China University of Mining and Technology, Beijing 100083, China; dr-xieq@cumtb.edu.cn

**Keywords:** pyrolysis behavior, mesophase pitch, ReaxFF MD, molecular structure, TG-FTIR/MS

## Abstract

Mesophase pitch is regarded as a profoundly promising candidate for the production of advanced carbon-based multifunctional materials such as carbon fibers, carbon microspheres, and carbon foams owing to its excellent intrinsic properties. Consequently, a deeper understanding of pyrolytic chemistry is indispensable for the efficient and environmentally friendly utilization of mesophase pitch. In this study, details about the structure compositions and microscopic morphologies of petroleum-driven mesophase pitch (pMP) were investigated through ultimate, FTIR, XPS, and ^13^C-NMR analyses. Furthermore, a large-scale molecular model of typical pMP with 11,835 atoms was constructed to unveil the comprehensive pyrolysis behaviors and the underlying reactions. Significantly, the evolution of specific chemical bonds and the decomposition of crucial molecular fragments were elucidated within an amalgamation of experimental TG-FTIR/MS and ReaxFF MD simulation. Accordingly, three fundamental reaction stages were artificially divided, including the low-temperature reaction, rapid thermal decomposition, and the molecular condensation reaction. During the rapid thermal decomposition stage, the cleavages of C–C and C–O bonds cooperatively contributed to the formation of C_2_H_4_ and H_2_O gaseous products. As the temperature escalated to the molecular condensation stage, the pyrolysis process was governed by the dehydrogenation condensation, accompanied by an augmentation of C–C and H–H bonds and a diminution of C–O and C–H bonds. Additionally, the rare graphitization phenomenon was observed, suggesting a remarkable degree of structural organization in pMP. Overall, the results of ReaxFF MD simulations complement experimental observations, successfully reproducing the microstructure of pMP and atomic-scale pyrolysis behavior, thereby providing invaluable insights for the rational guidance of efficient utilization of pMP and other related carbonaceous precursors.

## 1. Introduction

Mesophase pitch, also known as mesophase asphalt, is esteemed for its distinctive micro-morphology, characterized by structured entities presenting in stratified and/or filamentous arrangements of carbon atoms, imparting traits akin to graphite [1]. With its remarkable molecular capabilities, mesophase pitch has emerges as a profoundly promising candidate for advanced carbon-based multifunctional materials, including carbon fiber, carbon microspheres, and carbon foam [2,3,4,5]. As a results, it has garnered significant attention in cutting-edge fields such as thermal management, energy storage, catalysis, and electrode applications [6,7,8,9]. Actually, the thermal decomposition of mesophase pitch in an inert atmosphere or vacuum stands as the fundamental procedure for manufacturing carbon-based multifunctional materials, with reactions serving as pivotal events in the processes of carbonization, activation, and combustion conversion [10]. Despite the well-established recognition of mesophase pitch as a crucial carbonaceous foundational material, regrettably, scant attention has been directed towards elucidating its pyrolysis behavior or mechanisms in published articles. Considering the diverse origins (categorically encompassing coal, petroleum, and naphthalene) and varied structural composition (such as various oxygen-containing functional groups), which give rise to a complex reaction environment, a deeper insight into the pyrolysis chemistry is indispensable for achieving high efficiency and environmentally friendly utilization of mesophase pitch.

In general, the pyrolysis chemistry of mesophase pitch is commonly perceived as an enigmatic and intricate procedure that involves numerous fragmentations and rearrangement reactions of carbonaceous structures alongside abundant free radical intermediates generated [11]. The dissociation and integration of associated radicals exhibit high reactivity and have exceedingly brief lifetimes, rendering them difficult to capture experimentally [12], even with state-of-the-art experimental techniques. Undoubtedly, depicting the geometric evolution of mesophase pitch at the atomic level solely through experimental characterization presents daunting obstacles, given its susceptibility to temperature and temporal fluctuations, thereby affecting the accurate elucidation of the pyrolytic dynamics [13]. On the other hand, theoretical investigation provides a promising avenue for simulating the intricate chemistry and diverse reaction pathways involved in mesophase pitch pyrolysis systems. Particularly noteworthy is the utilization of reactive force field molecular dynamics (ReaxFF MD) methodology, which integrates molecular dynamics with reactive force field [14]. Indeed, due to its adept depiction of the dissociation and formation of chemical bonds, ReaxFF MD achieves accuracy comparable to density functional theory (DFT) with markedly enhanced simulation efficiency, thereby substantially reducing computational costs and the associated time investment [15,16]. Furthermore, by incorporating non-bonded interactions such as van der Waals and Coulomb interactions into the simulation category, ReaxFF MD is also suitable for dynamically describing chemical reactions in large reaction systems (>1000 atoms) without any predefined reaction pathways [17,18]. More remarkably, the feature of not requiring predefined reaction pathways renders it an appropriate and promising selection for simulating the complex pyrolysis system of mesophase pitch.

Recent advancements in scholarly inquiry have substantiated the efficacy of the ReaxFF MD methodology in reproducing the pyrolysis behavior and ensuing product distribution of carbonaceous precursors across varied thermal conditions [19,20,21,22,23,24,25]. For example, Zheng and colleagues have achieved groundbreaking outcomes by aligning qualitative temperature profiles derived from ReaxFF MD simulations with empirical evidence from thermogravimetry experiments, thus pioneering the correlation between simulation outputs and tangible evidence through the discernment of covalent bond rupture [26]. This revelation heralds a promising avenue for harmonizing simulation results with real-world observations. In addition, You et al. employed the ReaxFF MD approach to reveal pivotal parameters of modified coal tar pitch, including true density, critical peak intensity of radial distribution function (RDF), sp^2^ hybridized bond ratio, and the proportion of six-membered ring, which exhibited a trend of initial decline followed by escalation with increasing temperature, consistent with the conclusions obtained by experimental detection methods such as X-ray diffraction (XRD) and small-angle X-ray scattering (SAXS) [27]. Additionally, Castro-Marcano has delved into the pyrolytic mechanism of larger size molecular structures, contending that the pyrolysis dynamics of Illinois no. 6 coal (over 50,000 atoms) are principally orchestrated by the liberation of hydroxyl groups and the dehydrogenation of hydroaromatic structures, followed by crosslinking and cleavage involving heteroatoms. The main pyrolysis products include hydrogen, methyl, ethylene, acetylene, formaldehyde, ethyl, alkyl phenol, and alkyl naphthalene, all in accordance with the experimental findings [28]. Moreover, the pyrolytic evolution of Liulin coal has been investigated by Zheng, revealing a systematic progression consisting of four principal stages delineated by the rupture of bridging bonds under controlled thermal conditions within ReaxFF MD simulations [29]. Although substantial progress has been made in understanding the pyrolytic chemistry of traditional carbonaceous precursors represented by coal, elucidating the pyrolysis behaviors of novel mesophase pitch remains a significant challenge that has confounded scientists for decades.

In this paper, we integrated the experimentally in situ TG-FTIR/MS with theoretical ReaxFF MD methodology to provide insights into the temperature-dependent distribution of product and chemical pathways that play critical roles in the in-depth investigating of pyrolytic properties of petroleum-driven mesophase pitch (pMP), which has not been reported, to our best knowledge. A series of ReaxFF MD simulations were meticulously executed, employing a structural model of pMP containing 11,250 atoms, constructed based on experimental characterizations including elementary analysis (EA), ^13^C-NMR, XPS, and FTIR. Subsequently, the simulation trajectories underwent scrupulous scrutiny to delineate the evolution of product profile and the underlying pyrolytic reaction mechanism. The authenticity and reliability of the computational outcomes were methodically evaluated through correlation with experimentally in situ TG-FTIR/MS analyses. Systematically, the methodologies employed in simulations including the construction of the pMP structure model, simulation parameters, and trajectory analysis techniques, as well as the experimental measurement methods, have been expounded upon in Section 2. In Section 3, we have comprehensively delved into discussions pertaining to the evolution of pyrolyzate profiles, the dissociation and polycondensation of chemical bonds, the pathways engendering main gaseous compounds, and their corresponding deliberations. The Section 4 succinctly summarizes the essence of this study.

## 2. Materials and Methods

### 2.1. Preparation of Sample

The commercial pMP sample, provided by Liaoning NOVCARB Carbon Materials Co., Ltd. in Liaoning, China, was selected for examination in this study. The sample underwent grinding and sieving procedures to attain a particle size of approximately 300 mesh. Subsequently, it was desiccated over a period of 24 h at a temperature of 373 K to eliminate residual moisture. No additional manipulations were conducted subsequent to this process. The ultimate analyses of the constituent components within the pMP are delineated in Table 1, ascertained through the utilization of the advanced analytical equipment, namely the Elementar Vario EL (Elementar, Langenselbold, Germany).

Where “Experimentally” and “Theoretically” represented these results derived from experimental measurements and theoretical simulations, respectively.

### 2.2. Experimental Methods

#### 2.2.1. C-NMR

The ^13^C solid-state nuclear magnetic resonance (^13^C-NMR) measurements were performed utilizing an Advance III 600 MHz instrument (Bruker, Bremen, Germany) with a 3.2 mm H/X/Y triple resonance probe. The gradient field strength exceeded 28 T/m, with the resonance frequency of the magnetic field at 600.13 MHz. Moreover, the cross-polarization duration and the relaxation delay period were set to 2 ms and 4 s, correspondingly.

#### 2.2.2. FTIR Spectrometer

Fourier transform infrared spectroscopy (FTIR) features were elucidated utilizing a Thermo Scientific Nicolet iS20 instrument (ThermoFischer, Waltham, MA, USA), equipped with an infrared laser emitter, operating across a spectral range from 4000 to 400 cm^−1^. The critical parameters were set at 30,000:1 (P-P) for signal-to-noise ratio and 2 cm^−1^ for spectral resolution.

#### 2.2.3. XPS Spectrum

The analysis via X-ray photoelectron spectroscopy (XPS) was executed utilizing a Thermo Scientific ESCALAB 250 Xi instrument (ThermoFischer, USA) with an X-ray excitation source of Al Kα (hν = 1486.6 eV), operating at a 14.6 kV voltage and a 13.5 mA filament current. The passing energy was configured to 20 eV with a step size of 0.1 eV, and charge correction was carried out using the binding energy of C_1s_ = 284.8 eV as the energy reference standard.

#### 2.2.4. TG-FTIR

The TG-FTIR instrument (Bruker TGA-IR/NEZTSCH STA 449 F5, Bielerica, MA, USA) was deployed for continuous monitoring of the relative mass variation and the specific composition of gaseous products. Throughout the experimental procedure, precise temperature control was upheld for both the inlet and outlet pipelines, as well as the gas reservoir, steadfastly maintained at 473 K. Additionally, argon (Ar) served as the protective atmosphere with a volumetric flow rate of 50 mL/min. The pyrolysis temperature underwent incremental escalation from ambient temperature to 1273 K, orchestrated at controlled heating rates of 5 K/min, 8 K/min, and 10 K/min. Otherwise, meticulous calculation of the derivative thermogravimetric (DTG) curve was executed to pinpoint the temperature corresponding to the maximum rate of pyrolysis reaction.

#### 2.2.5. TG-MS

The TG-MS instrument (thermo plus EVO_2_/ thermo mass photo, Waltham, MA, USA) was employed for the continuous surveillance of relative mass oscillations and the specific composition of the gaseous products. The pertinent parameters mirrored those employed in the TG-FTIR measurements.

### 2.3. Computational Methods

#### 2.3.1. Model Construction

**Figure 1:**
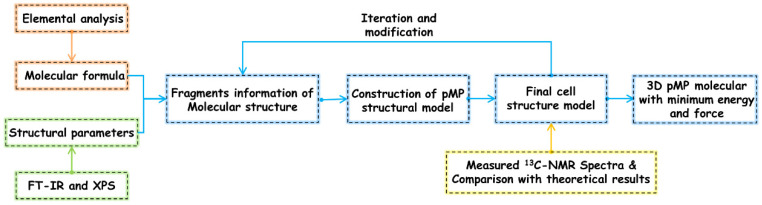
Procedure of three-dimensional model construction and optimization.

**Figure 2:**
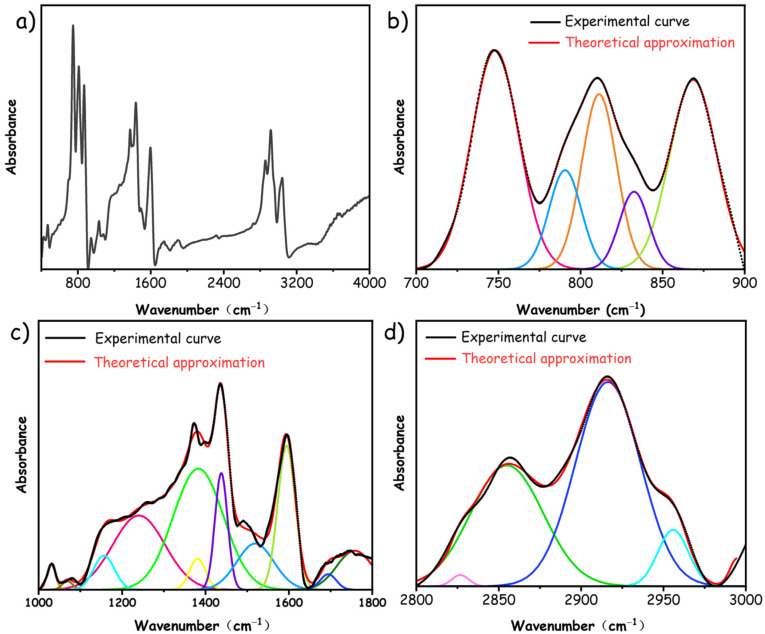
(**a**) FTIR spectra, Curve-resolution into different components, including (**b**) aromatic structure, (**c**) characteristic functional groups, and (**d**) aliphatic functional groups.

**Figure 3:**
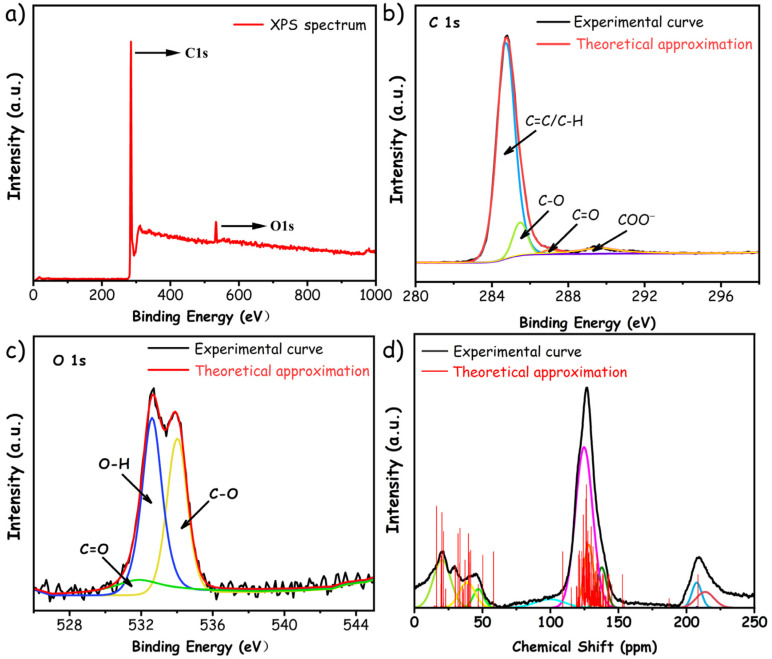
(**a**–**c**) XPS spectrum of pMP and its correlative curve-resolution into different elements, including (**b**) C 1s and (**c**) O 1s; (**d**) ^13^C-NMR spectra and its correlative curve-resolution of pMP.

**Figure 4:**
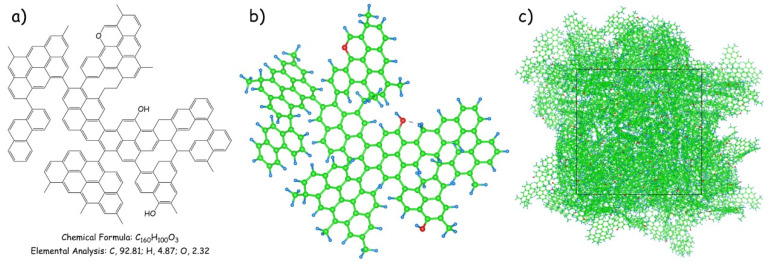
(**a**,**b**) Molecular structure unit and its stability-centric structural model of pMP; (**c**) 3D representation of the pMP molecule with a square area of 49.38 × 49.38 × 49.38 Å. The green, red, and blue balls represent carbon, oxygen, and hydrogen atoms, respectively.

The formulation of pMP molecular models adhered to the following fundamental principles (as illustrated in Figure 1): initially, the pertinent structural parameters, such as the details about elemental composition, proportions, and functional group information, were garnered from experimental characterizations; thereafter, based on these revelations, the molecular framework was constructed utilizing Materials Studio (MS 6.0) software, with optimization procedures executed to achieve energy and force minimization; finally, NMR measurements were employed to elucidate the average carbon skeletal structure, thereby substantiating the rationality of the fabricated pMP molecular models. According to the molecule fragments identified in Figure 2, Figure 3 and Figure 4, the molecular model with its three-dimensional periodic structure of corresponding pMP macromolecular system was constructed with the Amorphous Cell module in MS. This module employed a Monte Carlo approach to assembling molecules within a cell, while ensuring a realistic distribution of torsion angles in accordance with any given force field, thereby minimizing atomistic interactions. Moreover, the initial density of the molecular models was set at 0.5 g/cm^3^ to preclude the superposition of an aromatic ring structure during the model construction. The COMPASS force field and constant-pressure, constant-temperature (NPT with a certain number of particles (N), pressure (P), and temperature (T)) ensemble in the Forcite module were utilized to perform the dynamic simulations under high-pressure conditions (300 K, 100 Mpa) to rationalize the system equilibrium, followed by an annealing procedure utilizing the canonical ensemble (NVT with a certain number of particles (N), volume (V), and temperature (T)) to conduct structural energy optimization. The Berendsen approach was employed in both the NPT and NVT ensembles to control the temperature/pressure parameters, with the damping coefficient and time step configured at 0.1 ps and 1 fs, respectively.

#### 2.3.2. ReaxFF MD Simulation

ReaxFF MD simulations were performed using the Large-scale Atomic/Molecular Massively Parallel Simulator (LAMMPS, 28 March 2023), a classical molecular dynamics software. The simulations referred to a temperature range from 300 to 3000 K, employing various heating rates of 10 K/ps, 20 K/ps, 30 K/ps, and 40 K/ps, with a heating duration of 260 ps. In order to ensure precise initialization of the temperature, an initial isothermal progress of 10 ps at 300 K was implemented, followed by a total simulation time of 460 ps for each case. Additionally, the simulations were conducted under periodic boundary conditions, utilizing the NVT ensemble and Berendsen temperature control method with a damping coefficient set to 0.1 ps. The C/H/O/N/S reactive force field in ReaxFF MD was selected to facilitate the description of chemical reactions occurring during the pyrolysis of pMP. It is noteworthy that the simulated temperatures in ReaxFF MD significantly exceed those in the experiments, which arises from the inherent difference in timescale between simulations (picoseconds) and experiments (seconds) [17]. To promote the progress of chemical reactions of complex systems in a limited time, raising the simulation temperature can intensify the collision between atoms to accelerate the reactions [30,31]. Notably, while ReaxFF MD demonstrates remarkable proficiency in addressing complex chemical systems and reaction dynamics, it is inherently constrained, particularly in relation to the treatment of long-range interactions and charge transfer. To mitigate these deficiencies, we meticulously selected appropriate parameters for our simulations and cross-referenced our findings with experimental results to validate the results. Additionally, multiple iterations of the simulation were conducted to minimize potential errors. Furthermore, we referenced published articles to refine and substantiate the outcomes of our simulations. Overall, throughout the course of this study, we have implemented a comprehensive series of measurements both in the simulation process and in the analysis of the results to enhance the accuracy and reliability of our findings.

## 3. Results and Discussion

### 3.1. pMP Molecular Structure

In order to gain insight into the pyrolysis behavior of pMP, it is imperative to construct a molecular structure that is not only rational but also authentic. The experimental FTIR technique has been demonstrated to provide structural detail and insights into the inherent composition and functional groups for investigation of ReaxFF MD simulation of carbonaceous precursors [32,33]. Figure 2 elucidates the intricate structural parameters of pMP from the FTIR spectrum, categorized into three principal segments involving the aromatic structure (700–900 cm^−1^), characteristic functional group (1000–1800 cm^−1^), and aliphatic functional group (2800–3000 cm^−1^) [34]. At the region of 700–900 cm^−1^ (Figure 2b), four substitution patterns of benzene rings within pMP’s aromatic structure are identified: specifically, penta-substituted benzene (the main pattern constituting 33.77%), tetra-substituted benzene (30.28%), tri-substituted benzene (7.45%), and di-substituted benzene (28.5%), indicating a densely interconnected specification within the molecular structure of pMP, as listed in Table 2. Furthermore, the peak-fitting information associated with the characteristic functional groups at the region of 1000–1800 cm^−1^ is depicted in Figure 2c and Table 2. It reveals that the aromatic C=C vibrations accounted for 22.83%, underscoring benzene ring structures as principal components with abundant aliphatic hydrocarbons in the side chain. Stretching vibrations of oxygen-containing groups manifested in alkyl ether, aryl ether, and phenolic hydroxyl, with phenolic hydroxyl predominant at 20.82%. Additionally, details of the peak-fitting parameters for aliphatic functional groups are delineated in Figure 2d and Table 2. The C–H stretching vibrations of aliphatic hydrocarbons (mail methyl), situated at 2956 cm^−1^ and 2854 cm^−1^, comprised 56.39% of the total, suggesting their predominant presence among these functional groups.

In addition, XPS measurements have also been utilized to unveil the elemental composition and their corresponding percentage within pMP molecules, similar to the procedures conducted with other carbonaceous precursors [35,36,37]. Figure 3a–c depicts the XPS spectra and their correlative curve-resolution into different components. According to the results presented in Figure 3b and Table 3, it suggests that the molecular architecture of pMP is mainly composed of aromatic rings, as indicated by the notably higher proportion of C=C bonds (82.58%), which is consistent with the findings of FTIR spectroscopy. Otherwise, the diverse forms of oxygen species have been investigated, where phenolic hydroxyl is speculated to be the preferential functional group, supported by the calculated proportions of O–H (49.88%) and C–O (38.31%) depicted in Figure 3c and Table 3.

Actually, the detailed structural parameters derived from the FTIR and XPS measurements, coupled with insights referring to previous investigations into various pitch-based structural paradigms [38,39], facilitated the meticulous manual construction of the molecular structural model for pMP after gradual regulation and optimization, as displayed in Figure 4. Figure 4a delineates the structural configuration of pMP, showcasing how the presence of ether bonds (–O–) and bridge bonds (–CH_2_–CH_2_–) within the aromatic structure serve as interconnections to sequentially bind the secondary units. Therefore, it is reasonable that aliphatic methyl and oxygen-containing phenolic hydroxyl were the main substituent functional group entities along the side chain, deduced from the aforementioned observations. Additionally, a stability-centric structural model, characterized by energy and force minimization, was presented in Figure 4b. Notably, the constructed structure appeared relatively compacted, featuring small amounts of intramolecular hydrogen bonds contributing to heightened molecular stability. Remarkably, the three-dimensional representation of the pMP molecule, comprising 11,835 atoms and bearing the chemical formula C_7200_H_4500_O_135_, was ultimately attained by geometric optimization and dynamic annealing procedures, as shown in Figure 4c. Ultimately, the optimized pMP model manifested a density of 1.22 g/cm^3^, closely approximating the true density determined by experimental methodologies. Furthermore, the elemental compositions (as delineated in Table 1) of C (92.81), H (4.84), O (2.32), and C/H (1.59) were similar to the empirical analytical findings and published results [40].

Furthermore, the detailed carbon structural parameters of the pMP were determined using ^13^C-NMR analyses to verify the rationality of the constructed molecular model, as demonstrated in Figure 3d. This revealed the presence of two conspicuous peak assemblies in both experimental and theoretical curves, corresponding to the aliphatic carbon within the 0–60 ppm range and aromatic carbon within the 100–150 ppm range, respectively. In addition, the certain subtle peaks within the 60–90 ppm range indicated the relatively diminished content of oxygenated aliphatic carbon in pMP. Given the diverse array and intricate nature of carbon species in pMP, the corresponding structural parameters were calculated to better delineate the manifestation of various carbon atoms based on the chemical shift and peak area of NMR spectra. It is pertinent to note that the carbonyl characteristic peaks observed within the 200 to 250 ppm range in Figure 3d might be attributed to the interference of impurity, as no distinct C=O characteristic peaks were discerned in FTIR and XPS measurements and hence overlooked in the calculation and analysis of ^13^C-NMR. As elucidated in Table 4, the structural parameters derived from experiential NMR and theoretical molecular simulation were largely congruent for pMP, thereby effectively validating the accuracy of molecular models. The experimental characterization results of pMP provide an important foundation and constraints for the construction of a representative model for the following ReaxFF MD simulation.

### 3.2. Pyrolysis Behaviors of pMP

**Figure 5:**
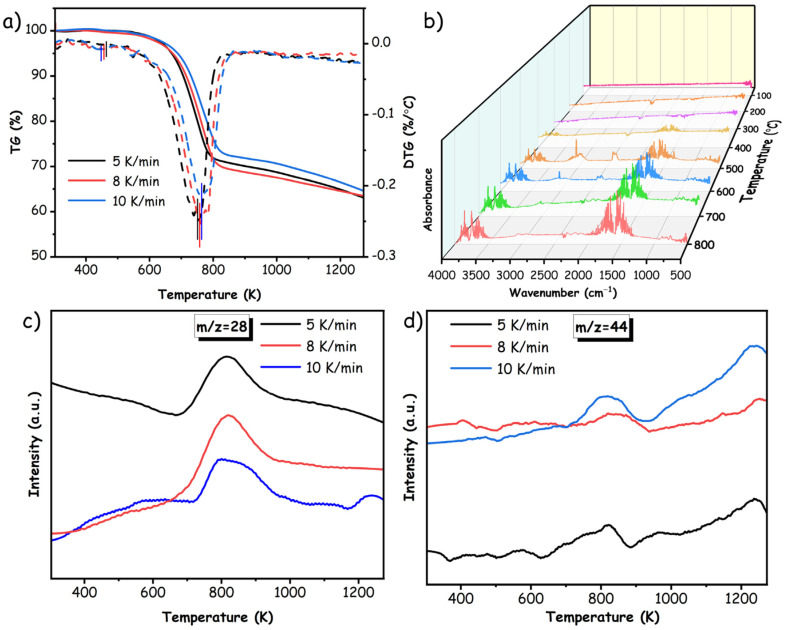
(**a**) The TG and DTG curves of pMP; (**b**) The temperature-dependent in situ TG-FTIF spectra during pyrolysis processes; (**c**,**d**) The in situ TG-MS spectra for representative C_2_H_4_ and CO_2_ products with various heating rates.

To elucidate the pyrolysis behavior of pMP, the TG-FTIR/MS spectrometers were deployed to continuously monitor the variations in gas constituents and functional groups throughout the pyrolysis procedure at varying rates of 5 K/min, 8 K/min, and 10 K/min, as demonstrated in Figure 5. It is well known that heating rates impact the pyrolysis yields and product distributions of carbonaceous precursors such as coal and biomass [41,42]. As depicted in Figure 5a, the onset of thermal degradation manifested at approximately 473 K, with the main decomposition process occurring at temperatures of 752 K, 758 K, and 765 K for the respective sample, accompanied by weight losses of 29.6%, 30.1%, and 26.5%, correspondingly. These negligible differences among the pyrolysis temperatures imply that the heating rates will slightly affect the intrinsic thermal properties of pMP, but rather predominantly manifest a substantial influence on the resultant decomposed products. In general, the decomposed products of pMP could be classified into three groups depending on carbon-containing atoms, including macromolecules (C_40+_), low molecular tar (C_5_~C_40_), and light gases (C_0_~C_4_) [43], wherein the gaseous products could subsequently volatilize/evaporate and be separated from the residues, offering insight into the pyrolysis behaviors of pMP. According to the FTIR observations corresponding to temperatures on the TG/DTG curves depicted in Figure 5b, the absorption bands of alkenes (mainly ethylene) located at 1520–1690 cm^−1^ and 620–760 cm^−1^ were discovered as the pyrolytic temperature reached 473 K, suggesting that a few bridge bonds and aliphatic hydrocarbons in the pMP molecular structure were susceptible to decomposition during the pyrolysis process. In addition, the oxygen-containing functional groups (ether, –O–) were deemed to favor the conversion to CO_2_, as evidenced by their imperceptible characteristic absorption bands at 2240–2400 cm^−1^ [44]. Thus, it was reasonable to attribute the weight loss (approximately 1.2%) at the initial pyrolysis stage to the decomposition of aromatic side chains, yielding small amounts of C_2_H_4_ and CO_2_ gaseous products.

**Figure 6:**
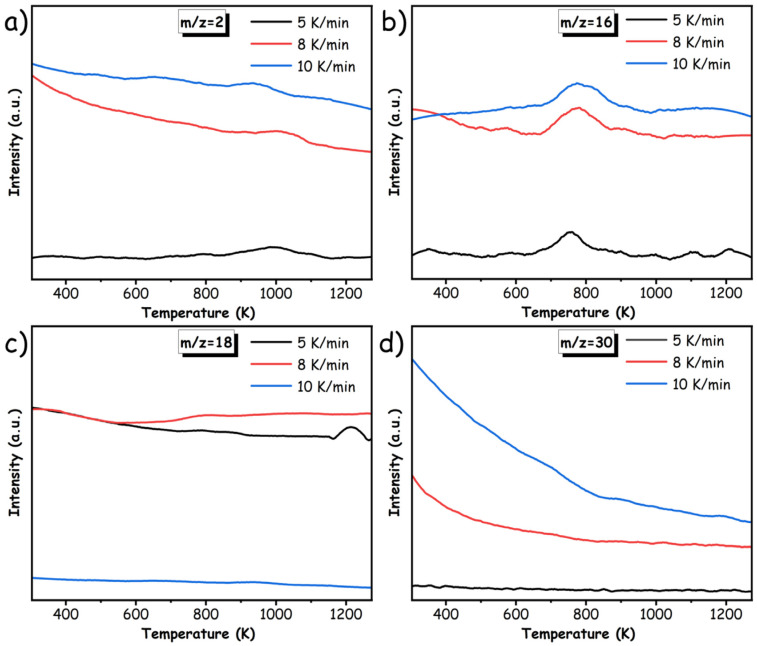
The in situ TG-MS spectra for representative products in pMP. (**a**) H_2_; (**b**) CH_4_; (**c**) H_2_O; (**d**) C_2_H_6_.

As the temperature ascended to 620–830 K, the peaks in the evolution of C_2_H_4_ and CO_2_ experienced a remarkable augmentation, profoundly contributing to the second weight loss stage. At this piont, a myriad of absorption bands appeared at the spectrum ranging from 2870–3120 cm^−1^ and 3620–3850 cm^−1^, corresponding to the characteristic stretching vibration of C–H and O–H groups, likely indicative of the CH_4_/C_2_H_6_ and H_2_O products. Notably, the potential evolutionary mechanism of CH_4_/C_2_H_6_ was identified as arising from the pyrolysis of aromatic methyl side chains, while the generation of H_2_O species originated from the transformation of oxygen-containing functional groups (mainly phenolic hydroxyl) as well as the re-reduction reaction of CO_2_ products. It is worth mentioning that the results gleaned from in situ TG-MS spectra indicated H_2_ as an additional conceivable gaseous derivative (as depicted in Figure 6), albeit absent in the FTIR spectrogram. As illustrated in Figure 5b, the peak intensity of C_2_H_4_ was significantly higher than others at this stage, suggesting its dominant role in evolution. As the temperature continued to rise, the peak intensities of C_2_H_4_ and H_2_O products exhibited a gradual augmentation, surpassing the thresholds of other products. This phenomenon can be attributed to the ongoing decomposition of stable oxygenated fragments and conjugated substituents at the high-temperature stage, facilitating the release of C_2_H_4_ and H_2_O gases. Conversely, the peak intensity of remaining products demonstrated a significant decline at higher temperatures, involving CH_4_/C_2_H_6_ and CO_2_, probably due to the diminishing decomposition of hydrocarbons and bridge bonds over time [45]. Additionally, the molecular polycondensation reaction of alkane fragments and the re-reduction reaction of CO_2_ moiety at high temperature also facilitate the release of C_2_H_4_ and H_2_O gases [46]. Hence, during the high-temperature stage, the escape of C_2_H_4_ and H_2_O gradually emerges as the dominant factor driving the weight loss of pMP.

In addition, the experimental in situ TG-MS technique was utilized to explore and identify the light gaseous products generated during the pyrolysis behavior of pMP. As depicted in Figure 5c,d and Figure 6, the characteristic signal corresponding to H_2_ (*m*/*z* = 2), CH_4_ (*m*/*z* = 16), H_2_O (*m*/*z* = 18), C_2_H_4_ (*m*/*z* = 28), C_2_H_6_ (*m*/*z* = 30), and CO_2_ (*m*/*z* = 44) were discovered as the gaseous product in pyrolysis proceedings. It is noteworthy that the potential presence of CO (*m/z* = 28) as a product was ruled out due to the absence of discernible characteristic peaks in TG-FTIR analysis (as illustrated in Figure 5b). Further experimental measurements were conducted with varying heating rates (5 K/min, 8 K/min, and 10 K/min) to thoroughly examine the influence of heating rates on the pyrolysis behavior of pMP. According to the findings, it indicated that variations in heating rates had minimal effect on the thermodynamic aspects (e.g., decomposition temperature) governing pyrolysis behavior, as substantiated by the uniformity of characteristic peaks observed in the resultant gaseous products. For example, the emergence of C_2_H_4_ species remained consistent at approximately 793 K across different heating-rate regimes. However, the heating rate determined the kinetic progression of gaseous products, thereby affecting their yield, as demonstrated in Figure 5c,d and Figure 6. Generally, there was an inverse relationship between the yields of C_2_H_4_/H_2_O and the rate of temperature increase, with a higher rate leading to decreased output. In contrast, other gaseous products (such as CH_4_) exhibited a positive correlation. This is likely attributed to the rapid heating rate impeding the subsequent molecular condensation of alkanes and re-reduction reactions of CO_2_ accompanying hydrogenation, resulting in limitations that reduced the production of C_2_H_4_ and H_2_O, while preserving the originally consumed gaseous species.

**Figure 7:**
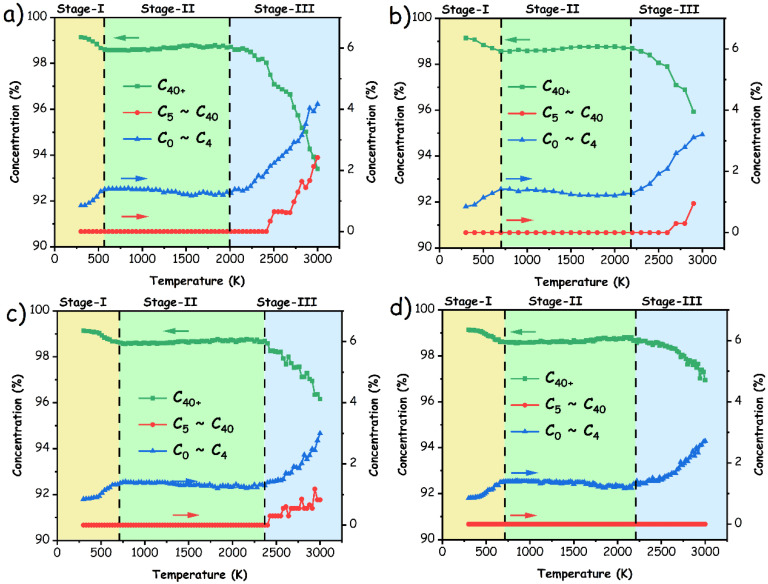
Evolutionary behaviors of associated pyrolysis products during the simulated pyrolysis procedures at various heating rates, including (**a**) 10 K/ps; (**b**) 20 K/ps; (**c**) 30 K/ps; and (**d**) 40 K/ps.

Although the aforementioned experimental measurements provided subjective insights into the pyrolysis behavior of pMP, the complexities of pyrolysis mechanisms, particularly the temperature-dependent atomic-scale structural alterations, remain a puzzle for scientists. To address this situation, we delved into theoretical ReaxFF MD simulations to enhance the understanding of overall product distribution on a nanoseconds scale. Figure 7 depicts the profiles of weight loss and major pyrolyzates with temperatures derived from ReaxFF MD simulation within the range of 300–3000 K at various heating rates of 10 K/ps, 20 K/ps, 30 K/ps, and 40 K/ps (denoted as pMP-10 K/ps, pMP-20 K/ps, pMP-30 K/ps, and pMP-40 K/ps). Taking pyrolysis behavior with a heating rate of 10 K/ps as an example, three representative stages were empirically delineated, wherein the amounts of C_40+_ fragments that were deemed as original samples at the initial decomposition stage I gradually decreased with increasing temperatures in the range of 300–550 K, concomitantly revealing the presence of light gaseous species (C_0_~C_4_). It was indeed consistent with the findings obtained from TG experiments in Figure 5a and demonstrated that ReaxFF MD simulation could reproduce pyrolysis processes of pMP to some extent, although the temperature range and quantitative values for weight loss were different between simulations and experiments. Upon further elevation of the simulated temperature, the proportion of the C_40+_ fragment exhibited nominal alteration during stage II (550–2000 K), subsequently plummeting sharply to yield low molecular tar (C_5_~C_40_) and gaseous products within the high-temperature ranges of stage III (2000–3000 K). This tendency in pyrolysis behavior was similarly observed in other cases, albeit causing an extension of the temperature range for each stage as the heating rate increased. For instance, at a heating rate of 40 K/ps, the initial decomposition reactions (stage I) occurred within the range of 300–800 K, surpassing the temperatures manifested at 10 K/ps. This phenomenon could potentially be ascribed to the accelerated reaction rates, leading to incomplete thermal decomposition processes that necessitate a prolonged reaction duration for completion. Moreover, the C_40+_ fragment at elevated temperatures was regarded as residual products, in which their concentration following the acceleration of the heating rate (93.4% in pMP-10 K/ps vs. 96.9% in pMP-40 K/ps) aligned with the experimental trends and verifies the rationality of the ReaxFF MD approach. Importantly, the increasing trends of large fragments at high temperatures were also detected from ReaxFF MD simulation to suggest the recombination reactions taking place frequently, which was not accessible by TG experiments. Overall, elevations in the heating rate may result in decreases in gas and tar production while simultaneously enhancing char output, which is most likely caused by an inadequate reaction within a short time period [47].

**Figure 8:**
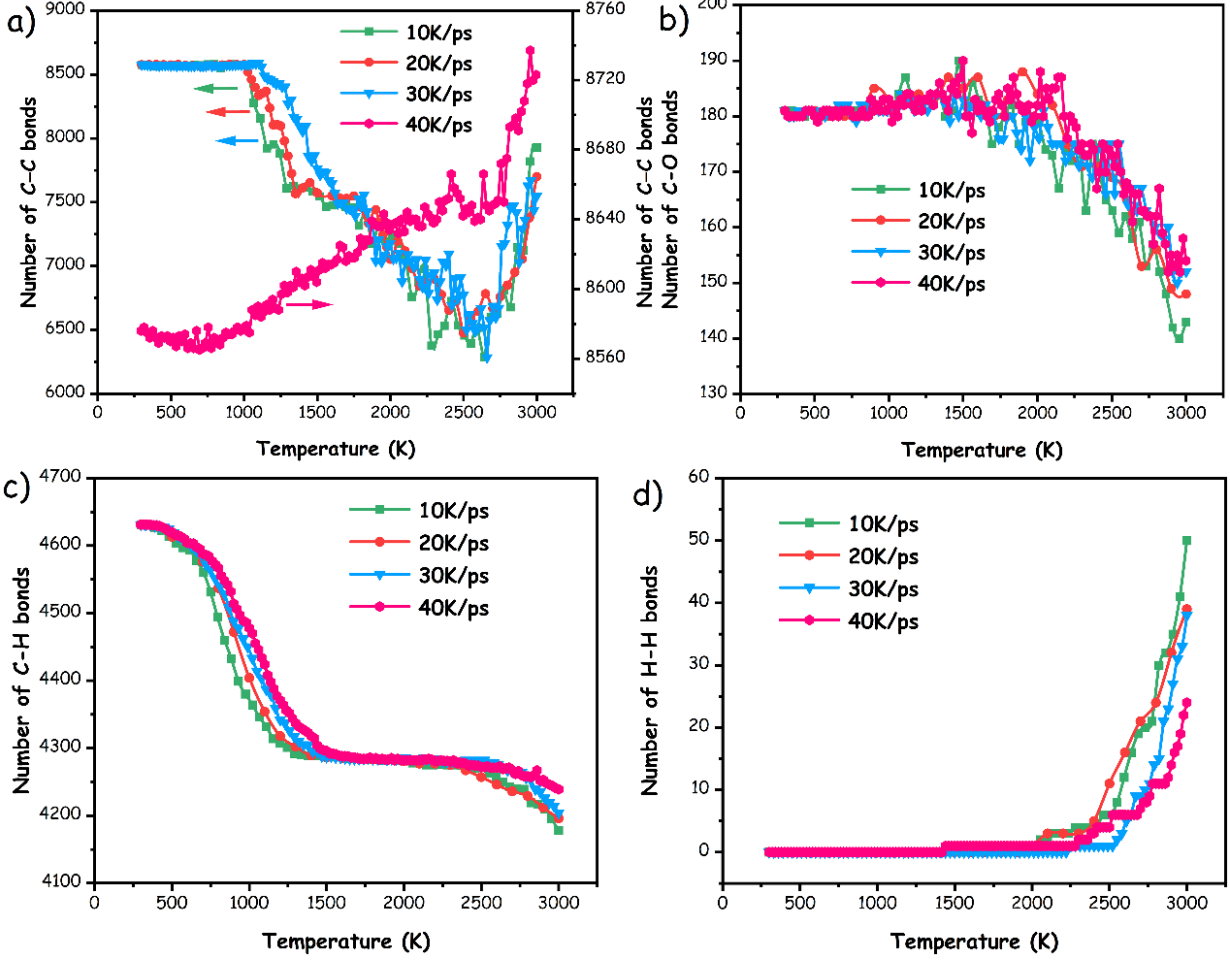
Evolutionary behaviors of associated chemical bonds during the simulated pyrolysis procedures at various heating-rate, involving (**a**) C–C bonds; (**b**) C–O bonds; (**c**) C–H bonds; and (**d**) H–H bonds.

Actually, each stage during the pyrolysis procedure of pMP represented the distinctive competitive reaction characterized by successive cleavage and recombination of chemical bonds. Figure 8 demonstrated the progression of associated chemical bonds throughout the pyrolysis processes. For all candidates, aside from the pMP-40 K/ps, the numbers of C–C bonds initially remained relatively constant with increasing temperatures (as displayed in Figure 8a) then exhibited a decrease followed by an increase, which is consistent with the variations of TG curves, corresponding to the low-temperature reaction, rapid thermal decomposition, and major condensation reaction, respectively. Otherwise, the amounts of C–C bonds in pMP-40 K/ps initially experienced a slight decrease before steadily increasing, indicating a relatively lower onset temperatures for the rapid thermal decomposition and condensation behavior. This could be construed as the rapid generation of highly active free radicals in a short time span, which would enhance the reactivity of the molecular fragments and accelerate the ensuing decomposition and condensation reactions.

According to the ReaxFF MD simulations depicted in Figure 8b, there was a slight augmentation in the numbers of C–O bonds within the system during the low-temperature region, probably attributable to the cyclization procedure undergone by certain oxygen-containing functional groups. The decomposition of oxygenated fragments (such as ether and phenolic hydroxyl) to release CO_2_ gas through the free radical mechanisms during the rapid thermolysis stage resulted in a marked decrease in the C–O bond content within the high-temperature region, a trend congruent with the in situ TG-FTIR findings presented in Figure 5b. Furthermore, the rate of decline in C–O bonds intensified as the temperature ascended from 2000 K to 3000 K. This occurrence was interpreted as the further depletion of synthesized CO_2_ gas and/or oxycarbide intermediates (e.g., –CO, –COH) in favor of hydroxide products (mainly H_2_O), thereby promoting a notable reduction in C–O bonds. Hence, with the continuous elevation in temperature, the predominant escape of H_2_O ensued in the consumption of oxygen species, a correlation also supported by the FTIR analysis.

Additionally, the evolution of C–H and H–H bonds during the pyrolysis were displayed in Figure 8c,d, respectively. A symbiotic relationship between the dissociation of C–H bonds and the formation of H–H bonds has been identified, wherein the amounts of C–H bonds decreased with rising temperature, while the H–H bonds exhibited a converse trajectory. In detail, the reduction in the number of C–H bonds in the initial temperature region resulted from the accelerated cleavage of unstable C–H bonds on the aliphatic side, facilitating the formation of unsaturated C–C bonds. Furthermore, the population of C–H bonds remained relatively stable as the temperature ascended to the rapid thermal decomposition stage, indirectly revealing that the rupture of unsaturated C–C bonds to yield alkene resultants predominated during this stage, as depicted in Figure 8a. This elucidated the theoretical origins of C_2_H_4_ formation observed experimentally. At relatively higher temperature region, the C–H bond experienced a conspicuous decline as the augmentation of the H–H bonds, signifying the prevalence of dehydrogenation condensation reactions under these conditions. Furthermore, taking the substantial reduction in C–O bonds into consideration, it implied that the H radicals exhibited a preference for undergoing polymerization with oxygen-containing intermediates (e.g., phenolic hydroxyl) to produce H_2_O molecules with enhanced efficiency, which helps to clarify the findings from TG-FTIR measurement that H_2_O serves as a critical resultant during the pyrolysis process. Interestingly, the evolution of these chemical bonds adhered to the principle of a positive correlation between residual value and heating rate, suggesting that excessively high heating rates may potentially engender incomplete thermal decomposition behavior.

**Figure 9:**
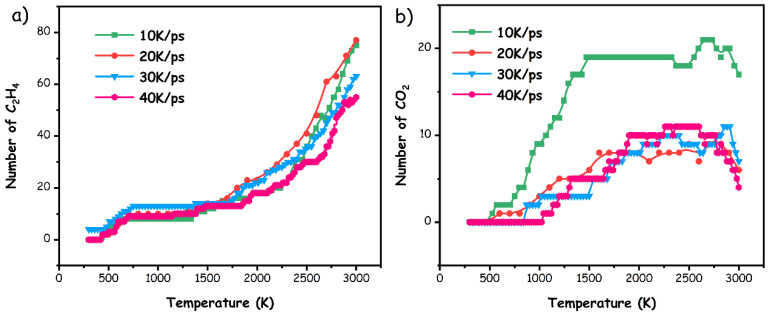
Evolutionary behaviors of associated light gases during simulated pyrolysis procedures at various heating rates, involving (**a**) C_2_H_4_ and (**b**) CO_2_.

As the principal volatile compounds produced during the pyrolysis of pMP, the alkene (predominantly C_2_H_4_) played a crucial role in determining the pyrolysis mechanisms. As depicted in Figure 9, the variations of C_2_H_4_ and CO_2_ species with the temperature were calculated by integrating the theoretical count of molecules in ReaxFF MD to validate the accuracy of the simulation. Accordingly, a mere trace of C_2_H_4_ and CO_2_ products were identified, aligning with the experimental observations originating from the initial decomposition of bridge bonds and aliphatic hydrocarbons in pMP. As the temperature increased, the number of both substances remained constant, which could be explained by the predominant cleavage of C–H bonds in this temperature region (as illustrated in Figure 8c), promoting the formation of C–O or unsaturated C–C bonds. At relatively higher temperatures, the production of C_2_H_4_ dramatically increased, while the concentration of CO_2_ decreased, in accordance with the experimental TG-FTIR outcomes. Meaningfully, the evolution trends based on the TG-FTIR/MS experiments and ReaxFF MD simulations were comparable with each other, in which the productivity of C_2_H_4_ species decreased with heightened heating rates, while the CO_2_ exhibited the opposite response.

**Figure 10:**
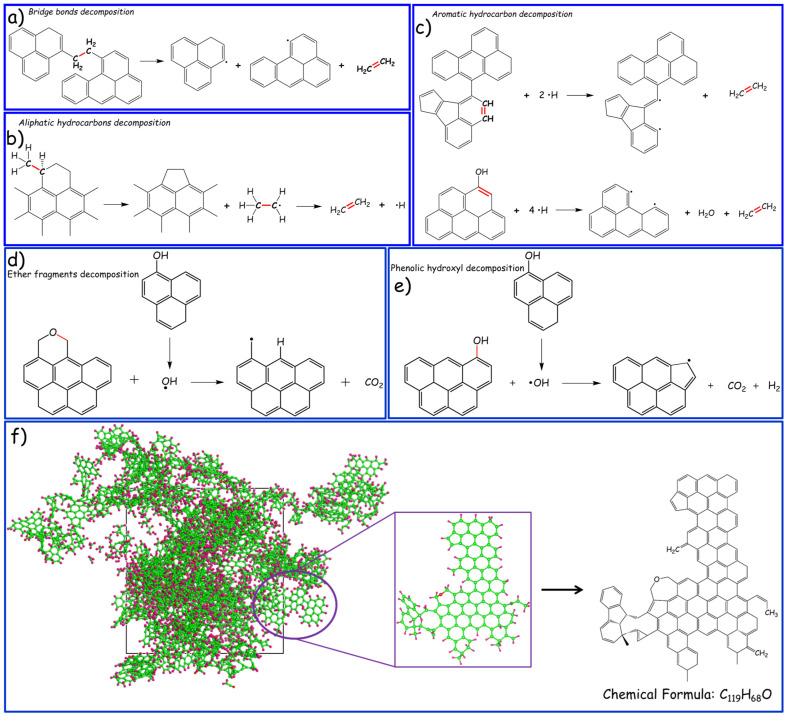
(**a**–**c**) The representative reaction pathways to yield the C_2_H_4_ product during the simulated pyrolysis procedure within the 10 K/ps heating rate; (**d**,**e**) The representative reaction pathways to yield the CO_2_ product during the simulated pyrolysis procedure within the 10 K/ps heating rate. Note, the surrounding structural geometry was ignored for simplification; (**f**) Molecular structure unit and its stability-centric structural model of pMP underwent ReaxFF MD simulation in 3000 K.

In order to unveil the evolutionary behaviors of microstructure configurations at varied temperatures at an atomic level, the VARxMD software visualization tool (V2.0 version) was utilized to analyze the simulation trajectories to obtain major product distribution and the involved reactions [48]. The representative reaction pathways observed through VARxMD, as depicted in Figure 10, have been enumerated here for the purpose of discussing the pyrolysis mechanism of pMP. Taking the C_2_H_4_ species as an example, the bridge bonds and aliphatic hydrocarbons favored to be cleaved to yield C_2_H_4_ gases at relatively low temperatures, while the decomposition of aromatic hydrocarbon decomposition and ring-opening processes preferred to be implemented as the temperature further increased, as depicted in Figure 10a–c. Furthermore, new covalent bonds, including carbon–carbon and carbon–heterogeneous bonds, have been documented, leading to the extension of a molecular skeleton of organic compounds and alterations in the chemical composition of alkylated compounds. Moreover, the oxygen-containing gaseous products, including CO_2_ and H_2_O, were also investigated to elucidate the intricate pyrolysis mechanisms of pMP, as illustrated in Figure 10d,e. According to the simulated findings, the oxygenated fragments derived from ether and phenolic hydroxyl groups preferentially decomposed to generate CO_2_ gas molecules during the pyrolysis process. At higher temperatures, the resultant CO_2_ and oxygen-containing functional groups readily interacted with hydrogen radicals to yield H_2_O species. This phenomenon is consistent with the evolutionary behavior of relevant chemical bonds, wherein the generation of CO_2_ during the pyrolysis process induces an increase in the number of C–O bonds, which are subsequently consumed as the temperature rises to produce H_2_O. It is noteworthy that the observed graphitization phenomenon within the ReaxFF MD simulation, wherein localized aromatic hydrocarbons exhibited a propensity to polymerize at high temperatures, gave rise to a microscale morphology resembling graphite layers with sustained skeletal alignment, as visualized in Figure 10f. This discovery, uncommon in other carbonaceous precursors such as coal, underscores a remarkable degree of structural organization in pMP. These revelations hold promise in addressing the present lack in understanding of pyrolysis mechanisms among diverse carbonaceous precursors at a molecular level, with particular relevance to pMP.

## 4. Conclusions

In this paper, an amalgamation of experimental methodologies and ReaxFF MD simulation was employed to construct the rational molecular structure of pMP and scrutinize the associated microscale thermal decomposition behavior, thereby gaining insights into the pyrolytic chemistry for the effective and environmentally friendly utilization of pMP. In particular, the investigations incorporate conventional FTIR, XPS, and ^13^C-NMR techniques to explore microstructure insights, as well as TG-FTIR/MS and ReaxFF MD methods to analyze the evolution of volatile distribution over time and varying temperatures. Accordingly, a large-scale molecular model of typical pMP with 11835 atoms was proposed to unveil the comprehensive pyrolysis behaviors and the underlying reactions, which have not yet been reported, to our best knowledge. The pyrolysis procedure of pMP has been classified into three stages depending to the temperatures, involving a low-temperature reaction, rapid thermal decomposition, and molecular condensation reaction, wherein the C_2_H_4_ and CO_2_ gaseous products were observed during the low-temperature region originated from the thermal degradation of aromatic side chains. In the high-temperature region, the C_2_H_4_ species predominate over others by several orders of magnitude, while the H_2_O products were identified as the main consumers of oxygen species in pMP. The VARxMD software (V2.0) visualization tool revealed that the dominant C_2_H_4_ product was mainly yielded by the decompositions of the bridge bonds and aliphatic hydrocarbons at relatively low temperatures, and the decomposition of aromatic hydrocarbon decomposition and ring-opening processes occurred at higher temperature in the rapid thermal region. Meaningfully, the observed graphitization phenomenon within the ReaxFF MD simulation underscores a remarkable degree of structural organization in pMP, which is uncommon in other carbonaceous precursors such as coal. Moreover, the consistent results between experimental techniques and ReaxFF MD simulations were obtained and all indicated that elevations in heating rate may result in a decrease in gas and tar production while simultaneously enhancing char output, most likely caused by inadequate reactions within a short time period. Overall, we have successfully reproduced the microstructure and atomic-scale thermal decomposition behavior of pMP through a combination of experimental techniques and ReaxFF MD simulations, offering valuable insights for the rational guidance of the efficient utilization of pMP and other associated carbonaceous precursors.

## Figures and Tables

**Table 1:** The constituent components (wt%) of pMP.

	C	H	O	C/H	Density (g/cm^3^)
Experimentally	93.44	4.42	2.14	1.75	1.28
Theoretically	92.81	4.87	2.32	1.59	1.22

**Table 2:** Infrared bands belonging to the aromatic structure.

Region	Center/cm^−1^	Width	Peak Area	Proportion	Assignment
Aromatic structure	748	33.98	3.00	28.50	di-substituted benzene
	790	23.16	0.92	7.45	tri-substituted benzene
	811	25.02	1.76	19.88	tetra-substituted benzene
	832	21.16	0.66	10.40	tetra-substituted benzene
	866	33.63	2.53	33.77	penta-substituted benzene
Characteristic functional groups	1030	23.91	0.24	1.15	C–O–C alkyl ether
	1068	30.40	0.10	0.49	C–O aryl ether
	1156	58.97	0.80	3.82	C–O aryl ether
	1240	147.95	4.33	20.82	C–O phenols
	1382	187.88	7.36	35.35	CH_3_–Ar
	1438	35.72	1.64	7.91	CH_3_–, CH_2_–
	1519	104.65	1.91	9.19	Aromatic C=C
	1592	49.84	2.84	13.64	Aromatic C=C
	1694	45.41	0.28	1.34	Aromatic (C=O)
	1758	101.26	1.31	6.29	Aliphatic (C=O)
Aliphatic functional groups	2826	12.07	0.03	7.22	Sym. R_2_CH_2_
	2854	51.63	1.37	55.59	Sym. RCH_3_
	2916	46.36	2.08	36.39	Asym. R_2_CH_2_
	2956	21.90	0.27	0.80	Asym. RCH_3_

**Table 3:** XPS C 1s and O 1s spectra and curve-resolution into different components.

Species	Binding Energy (eV)	Attribution	Proportion %
C 1s	284.8	C=C/C–H	82.58
	285.7	C–O	9.53
	287.1	C=O	1.32
	289.6	COO^−^	6.57
O 1s	531.8	C=O	11.81
	532.6	O–H	49.88
	534.0	C–O	38.31

**Table 4:** Structural parameters of pMP sample obtained from ^13^C-NMR.

Parameter	$f_{a}$	$f_{a}^{C}$	$f_{a}^{\prime }$	$f_{a}^{H}$	$f_{a}^{N}$	$f_{a}^{P}$	$f_{a}^{S}$	$f_{a}^{B}$	$f_{al}$	$f_{al}^{*}$	$f_{al}^{H}$	$f_{al}^{O}$
Experimentally	87.22	0.00	87.22	31.51	55.71	1.29	16.17	37.75	12.78	7.36	5.42	0.00
Theoretically	88.12	0.00	88.12	35.00	53.12	1.87	15.62	35.63	11.88	5.63	6.25	0.00

Parameters: $f_{a}$—total aromatic carbon; $f_{a}^{C}$—carbonyl; $f_{a}^{\prime }$—in an aromatic ring; $f_{a}^{H}$—protonated and aromatic; $f_{a}^{N}$—nonprotonated and aromatic; $f_{a}^{P}$—phenolic or phenolic ether; $f_{a}^{S}$—alkylated aromatic; $f_{a}^{B}$—aromatic bridgehead; $f_{al}$—total aliphatic carbon; $f_{al}^{*}$—CH_3_; $f_{al}^{H}$—CH or CH_2_; $f_{al}^{O}$—bonded to oxygen. “Experimentally” and “Theoretically” represent these results derived from experimental measurements and theoretical simulations, respectively.

## Data Availability

The original contributions presented in the study are included in the article, further inquiries can be directed to the corresponding author.
